# CNN Training with Twenty Samples for Crack Detection via Data Augmentation

**DOI:** 10.3390/s20174849

**Published:** 2020-08-27

**Authors:** Zirui Wang, Jingjing Yang, Haonan Jiang, Xueling Fan

**Affiliations:** State Key Laboratory for Strength and Vibration of Mechanical Structures, Xi’an Jiaotong University, Xi’an 710049, China; wangzirui@stu.xjtu.edu.cn (Z.W.); jing_jing_y@mail.xjtu.edu.cn (J.Y.); jianghaonan757@163.com (H.J.)

**Keywords:** crack detection, deep learning, data augmentation, small samples

## Abstract

The excellent generalization ability of deep learning methods, e.g., convolutional neural networks (CNNs), depends on a large amount of training data, which is difficult to obtain in industrial practices. Data augmentation is regarded commonly as an effective strategy to address this problem. In this paper, we attempt to construct a crack detector based on CNN with twenty images via a two-stage data augmentation method. In detail, nine data augmentation methods are compared for crack detection in the model training, respectively. As a result, the rotation method outperforms these methods for augmentation, and by an in-depth exploration of the rotation method, the performance of the detector is further improved. Furthermore, data augmentation is also applied in the inference process to improve the recall of trained models. The identical object has more chances to be detected in the series of augmented images. This trick is essentially a performance–resource trade-off. For more improvement with limited resources, the greedy algorithm is adopted for searching a better combination of data augmentation. The results show that the crack detectors trained on the small dataset are significantly improved via the proposed two-stage data augmentation. Specifically, using 20 images for training, recall in detecting the cracks achieves 96% and $F_{ext}^{\left(0.8\right)}$, which is a variant of F-score for crack detection, achieves 91.18%.

## 1. Introduction

Cracks are commonly one of the most dangerous defects in structures, such as bridges, pressure vessels, mining equipment, aero-engines, etc. Any crack on the key components of these structures can lead to accidents. Thus, it is necessary to monitor the integrity of structures and evaluate the crack for safety [1,2,3,4]. To date, manual visual inspection is the primary and most widely used method in structure integrity monitoring, and it is expensive and time-consuming [5,6]. Furthermore, the accuracy of detection mostly depends on the experience and attention of technicians, and the cracks are easy to be missed. Hence, to achieve efficient and reliable inspection, developing automatic methods to detect cracks is of paramount importance.

To date, there are many methods of crack automatic detection. Most studies used manual approaches for feature extracting from images, which possess poor robustness [7,8]. Recently, deep learning was successfully applied to image recognition [9,10,11]. On this basis, some methods of crack detection based on convolutional neural networks (CNN) are proposed [12,13,14]. Cha et al. [14] proposed a defect detection method based on Faster R-CNN [15]. They obtained fine results but with an image split strategy, which loses some positioning capacity of the detector. A similar method for crack detection is also discussed by Li et al. [16]. They identified that the crack detector is underestimated by the mean average precision (mAP) method, which is a widely used standard for general object detection [17]. Additionally, they found that this underestimation is caused by the random fractal characteristics of cracks, and a fair evaluating method named covering evaluation (CovEval) is proposed.

However, to obtain excellent performance, a large amount of data is required in the network training, because in nature, all deep learning models are established on the data [18,19]. In practice, due to the commercial confidentiality of data, it is difficult to obtain thousands of data for CNN training. This presents a challenge to object detection of discovering a means to train a reliable neural network model with a few images. Data augmentation is a common strategy to achieve a better performance on small datasets. It generates new samples by transforming the original data. This process is proved to benefit the model in generalization ability and robustness [20]. By various data augmentation methods on images, such as random cropping, rotation, radiation transformation, and Gaussian noise, the limited samples are more fully used to train better models for practices [21,22,23]. Furthermore, the best data augmentation strategy is dataset-specific. This means that the effectiveness of different data augmentation methods varies greatly for different datasets. Therefore, it is worthwhile to study the data augmentation of crack detection.

On the other hand, the trained models can also use data augmentation to acquire better detection in the practical inference process. This is a severe challenge in industrial practices, as once a crack, whose length exceeds the critical value, is missing, it can lead to a catastrophic accident. Technically, by augmenting images to be detected into multiple images, the trained models can obtain more than one chance to make the detection and identify any missing targets when processing an image. This trick is a performance–resource trade-off that can be a useful approach facing the challenge of reliability in practices.

To this end, we propose a two-stage data augmentation method. Stage one focuses on exploring the most effective data augmentation method in model training for crack detection. In our implementation, nine different data augmentation methods are discussed. The rotation method shows a significant improvement in experiments. The stage two is designed for the trained models in the inference process. The image to be detected is augmented for repetitive detection to improve the recall, which is crucial for reliable automatic industrial inspections like crack detection. To fit the limited resources in real practices, the greedy algorithm [24] is used to search for the most effective combination of data augmentation methods. With the two-stage method proposed, the performance of model trained on the small dataset is improved significantly. This work provides a practical guidance to the network training for crack detection in applications with only a small dataset.

The main contributions of this work are as follows:

1. By conducting experiments of various methods, the rotation method as a simple geometric transformation is found as the most effective data augmentation method in model training for crack detection.
2. The data augmentation is also employed for the inference process, and the greedy algorithm is successively applied to search for effective strategies.
3. A practical method for data augmentation comprises the two stages proposed for network training on a small dataset. When applying this method to train and deploy crack detectors, the recall of our best model reached 96% with only 20 images for training.

This paper is organized as follows: in Section 2, some related works regarding data augmentation are reviewed. In Section 3, we describe the methods used for augmenting the crack dataset. The details about experiments are shown in Section 4. In Section 5, we analyze and discuss the results. Finally, we conclude this paper in Section 6.

## 2. Related Work

Data augmentation is the most common practice in training CNN. Most of the existing research used geometric or photometric transformation methods to design the best augmentation strategies. For example, on ImageNet and some other natural image datasets, random cropping and adding noise were proved to be effective [9]. For periocular authentication, seven different photometric transformation methods are employed for data augmentation [21]. On a coarse-grained dataset, Taylor L et al. used six data augmentation methods that include clipping, rotation, flipping, color jittering, edge enhancement, and fancy Principal Components Analysis (PCA) [22]. For wire defect recognition, Tao X et al. investigated four data augmentation methods, and rotation was deemed most effective method [23]. On Common Objects in Context (MS COCO) dataset, Kisantal M et al. proposed a data augmentation method to improve the accuracy by oversampling images with small objects [25]. Various methods are designed for different datasets/tasks in these works, which mean that the method for data augmentation is dataset-specific.

Recently, Google’s team designed a strategy to automatically search for suitable data augmentation methods in different datasets. However, it is very costly as 15,000 Graphics Processing Unit (GPU) hours are required for searching for the best augmentation method [26]. With the development of generative adversarial networks (GANs) [27], some studies used GANs to generate additional samples for data augmentation, and they can produce a large amount of labeled data without losing category information [28,29,30]. However, training generative adversarial networks requires a lot of labeled data, thus, it is difficult to apply them to a small dataset. Moreover, by comparison with these intelligent methods, though manually designed methods require expert knowledge, they are effective and more practical.

Altogether, these literatures show that data augmentation can improve the performance of the model, and the best augmentation method is dataset-specific. However, there is no study on data augmentation for crack detection, and it is difficult to obtain a significant amount of data for it in the industry. This is what motivates us to develop a new data augmentation strategy for crack detection.

## 3. Methodology

**Figure 1:**
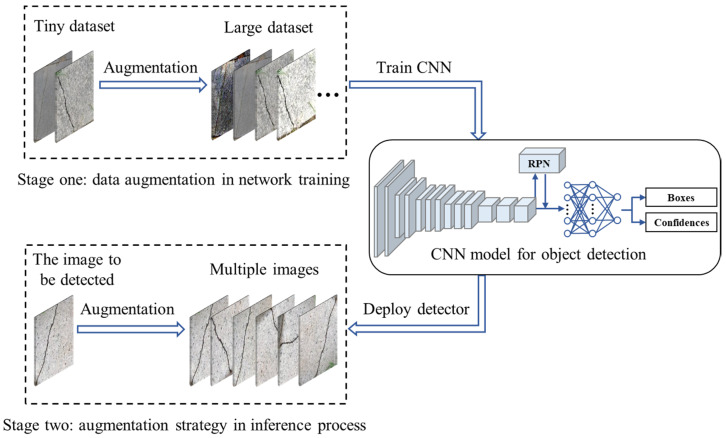
The completed process of our two-stage method.

We propose the data augmentation strategy from two stages: network training and the model inference process. Firstly, the rotation method, which outperforms all nine candidate methods, is used for data augmentation in the network training to improve the model. Then, a combined augmentation strategy, which is searched by the greedy algorithm, is employed in the inference process to achieve a further improvement for the trained models. The complete process of our method is shown in Figure 1. More specific details are shown in Section 3.1 and Section 3.2.

### 3.1. Stage One: Data Augmentation in the Network Training

Data augmentation generates new samples by inflating the original data artificially, and this process can be expressed as:

(1) $$D_{i}=A\left(D_{0},\left\{\theta_{i}\right\}\right)$$

where $D_{0}$ represents the collection of original raw images, A is a combination of specific data augmentation transformations, $\theta_{i}$ represents the parameters for data augmentation, and $D_{i}$ represents all the images generated by transformation.

The multiple of the augmentation target number and the original number in the crack dataset is described by the data augmentation factor f as:

(2) $$f=\frac{\left|D_{i}\right|}{\left|D_{0}\right|}$$

After data augmentation, all images $D_{i}$ are used for crack detector training, and the effect of each data augmentation method is evaluated.

**Figure 2:**
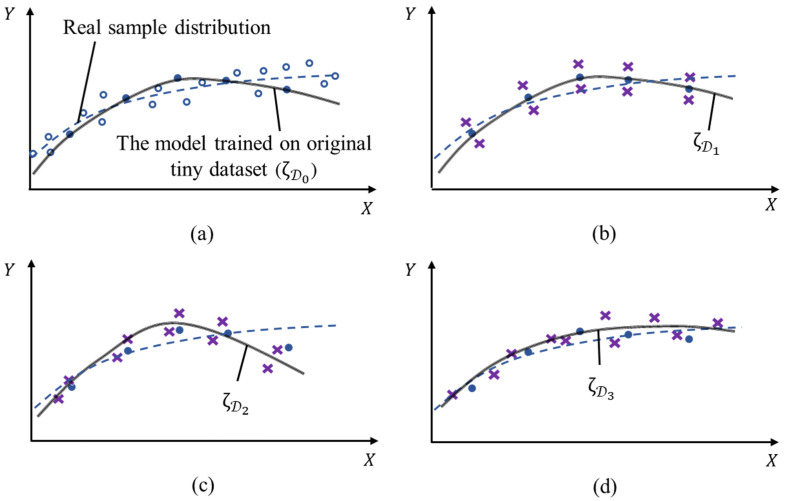
Schematic diagram of the effect of data augmentation on the trained models. (**a**) Comparison between real sample distribution and the model trained on the original small dataset. (**b**–**d**) Models trained with the augmented dataset via different data augmentation methods. ● represents samples in the small dataset; ○ represents real samples not collected; × represents the generated samples by data augmentation; the black lines are the trained models; the dashed line is the expectation of real sample distribution. Best viewed in color.

We briefly describe how the model gains via the various data augmentation. An example is given in two-dimensional space to illustrate the machine learning/deep learning process in high-dimensional space. Figure 2 plots models (or the real sample distribution) with curves. The model that is trained on dataset D is written as $\zeta_{D}$. $D_{0}$ represents the original small dataset. $D_{i}=A_{i}\left(D_{0},\;\left\{\theta_{i}\right\}\right)$ represents the dataset obtained from $D_{0}$ by the augmentation method $A_{i}$. In Figure 2a, $\zeta_{D_{0}}$ can not fit well with the real sample distribution ζ due to the insufficiency of samples. Based on the few original samples, the models in Figure 2b–d are trained with the augmented datasets $D_{1}$, $D_{2}$ and $D_{3}$ via augmentation methods $A_{1}$, $A_{2}$ and $A_{3}$. They have different influences on the final model, which may be positive, negative or no effect. In Figure 2b, when the generated samples are orthogonal to the original samples, $\zeta_{D_{1}}$ is almost similar to $\zeta_{D_{0}}$, which means that the samples generated by the data augmentation gain nothing for the model. In Figure 2c, the generated samples cause the model change against the trend of real distribution and obtain an unsatisfactory performance. The opposite situation appears in Figure 2d. The generated samples are more inclined follow the trend of real distribution, which improves the performance of the model $\zeta_{D_{3}}$. These analyses show that the best data augmentation method is dataset-specific, and for the specific task, different data augmentation methods affect the models quite diversely.

**Figure 3:**
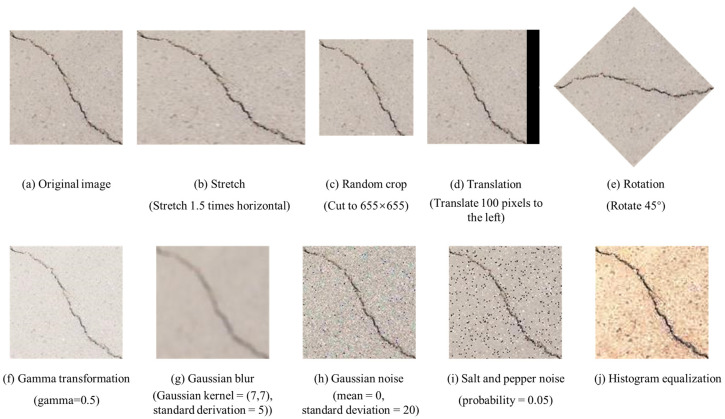
Nine different types of data augmentation methods. (**a**) Original image; (**b**–**e**) Four geometric transformations; (**f**–**j**) five photometric transformations.

To investigate the performance of different data augmentation on crack detection, nine types of data augmentation methods were adopted to generate new crack images, including four geometric transformations and five photometric transformations. As shown in Figure 3, all the data augmentation methods are as follows. The basic principles of each method are also listed.

- Horizontal and vertical stretch: stretch the images horizontally or vertically by a certain factor.
- Random crop: cut the images randomly according to the size of 655 × 655.
- Translation: shift the images 100 pixels in the X or Y direction.
- Rotation: rotate the images at an angle uniformly between 0° and 360°.
- Gamma transformation: correct the image with too high or too low gray, and enhance the contrast.
- Gaussian blur: reduce the difference of each pixel value to blur the image.
- Gaussian noise: add the noise whose probability density function follows Gaussian distribution.
- Salt and pepper noise: randomly add a white dot (255) or a black dot (0).
- Histogram equalization: enhance images contrast by adjusting image histogram.

### 3.2. Stage Two: Augmentation Strategy in the Inference Process

**Figure 4:**
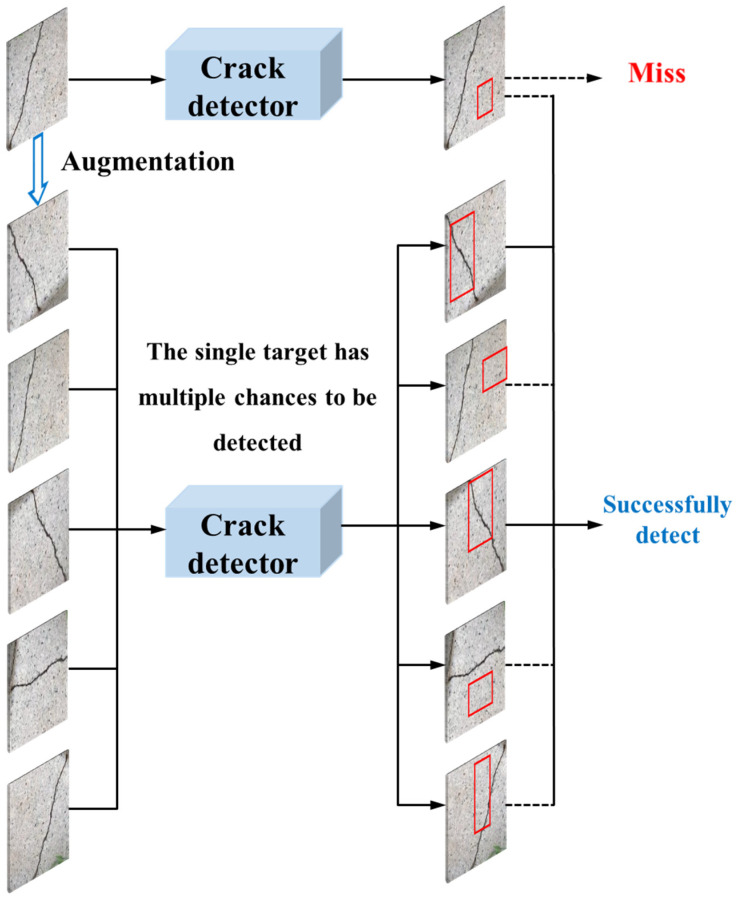
Data augmentation in the inference process. The crack obtains multiple chances to be detected in multiple augmented images when processing an actual image. If one crack is detected in any image after data augmentation, it is considered to be detected successfully.

To improve the recall of crack detection and realize a reliable detection, stage two focuses on the inference process of the trained models deployed in practices. Concretely, we augment one image to multiple images by various data augmentation methods, and then the single crack has multiple chances to be detected in multiple images to avoid being missed. Figure 4 shows the data augmentation in the inference process. However, this trick leads to an endless demand for resource in the inference process of the trained models, because giving the model more chances for detection, the recall of the targets can improve. With limited resources in practice, the trained models only have a few chances on one image for real-time detection. Thus, how to make full use of these chances and find the most effective augmentations becomes the key for a trained model to perform better.

Since there are numerous possible variants of one image via different data augmentation methods, the problem of combinatorial optimization must be addressed to identify more effective strategies:

(3) $$\{\begin{array}{c} \underset{A_{p}\in A}{\max}F\left(A_{p}\right)\\ s.t.\;\left|A_{p}\right|\leq p \end{array}$$

where $A=\left\{a_{1},a_{2}\ldots ,a_{n}\right\}$ denotes the collection of all n candidate methods for data augmentation; $A_{p}$ is a subset of A, and it denotes the collection of all selected method $a_{i}$ for data augmentation. $F\left(A_{p}\right)$ is the shorthand of $F\left(M\left(A_{p}\right)\right)$. It denotes the evaluating score of the model M which is trained on the training dataset augmented by methods in $A_{p}$. p is the maximum number of repetitive detection according to the real resources.

The exhaustive method is common for seeking the optimal solution for the above formula, but there are $C_{n}^{p-1}$ possible combinations to choose p−1 methods from n candidates for data augmentation, and it costs O($n^{p-1}$) tests to find the best combination. Obviously, as n increases, the number of required tests increases exponentially. It is costly in time and computation to obtain the optimal solution. We attempt to solve this problem with the greedy algorithm [24], which is the most common algorithm to solve this problem, and it costs O(n) to find the augmentation combination. Specifically, the greedy algorithm does not consider the global optimization, but selects the local optimal solution in each step and adds it to final solutions being constructed. In this work, the Algorithm 1 is as follows:
**Algorithm 1.** Greedy Algorithm in Model Inference ProcessInput: $A=\left\{a_{1},a_{2}\ldots ,a_{n}\right\}$;
$A_{p}=\left\{I\right\}$;Output: $A_{p}$For i = 2; i ≤ p doCompute all $F\left(A_{p}+\left\{a_{j}\right\}\right)$ for $a_{j}$ in A;Get $a_{j}$ that have $\underset{a_{j}}{\max}F\left(A_{p}+\left\{a_{j}\right\}\right)$;$A_{p}=A_{p}+\left\{a_{j}\right\}$;$A=A-\left\{a_{j}\right\}$;endreturn $A_{p}$;

Although the result is not the global optimal solution, it will be close to it, and when compared with the exhaustive method, it takes less time.

## 4. Experiment Settings

In this section, we introduce the experiment settings in detail, including the dataset, the CNN architecture, the training settings, and the evaluation method.

### 4.1. Dataset

**Figure 5:**
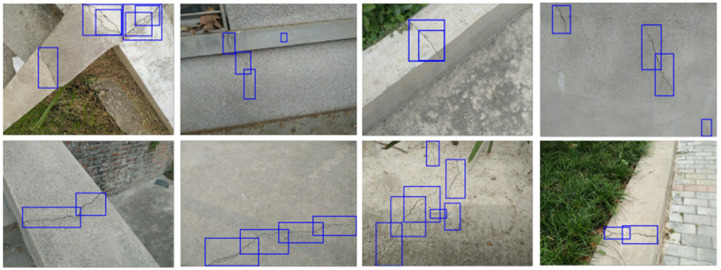
Crack images with their marks.

We collected 400 typical images of a crack from routine civil construction. Of the collected images, 320 images of them form the training set, and the other 80 images form the test set. All the images are photographed and marked by our team. Considering the random fractal characteristic of a crack, crack objects are marked with a special technique: long cracks are marked by several boxes with different scales instead of using one large box stretching across the whole image. In total, more than 2000 boxes are marked in ground truth (GT). As shown in Figure 5, crack images come from different environments: tiled pavement, asphalt road, concrete structure, marble tile, etc. The various data sources ensure that the crack detector can obtain the universal features of the crack.

### 4.2. Architecture for Crack Detection

Faster R-CNN [15] is used to detect and locate the crack directly in images. It is an object detection algorithm developed on RCNN [31] and Fast R-CNN [32]. A brief review of it is given here. Faster R-CNN mainly consists of three parts: a CNN backbone, a region proposal network (RPN), and a classifier. In this work, we adopt Visual Geometry Group Network (VGG) 16 as the backbone [33]. In the inference process, the inputted images in the form of a 3D tensor are initially processed into feature maps by VGG16. Then, RPN takes the feature map as an input and uses anchors that have nine different bounding boxes to locate the objects. Continuously, RPN identifies suspicious boxes as proposals among all default bounding boxes. Ranked by confidence from RPN, the region of interests set (RoIs) is formed by the top N proposals, and the corresponding vectors in feature map are sent into the softmax classifier after RoI pooling. Finally, the classifier obtains the final detection results. Further details regarding Faster R-CNN can be found in Ren’s paper [15]. Practically, we use the pre-trained VGG16 model that trained on the ImageNet dataset, and then fine-tuned the network on the crack dataset so that the crack detector can obtain a better performance. The weight parameters in the first several layers of the pre-trained model express low-level feature information such as contour or texture, and we aimed to retain the rich low-level feature learned from a large number of images in ImageNet. Thus, we freeze the convolution layer parameters of the first four layers in the fine-tuning process.

### 4.3. Training Settings

All experiments are run on one NVIDIA TITAN 2080Ti GPU. The codes are based on the TensorFlow [34] deep learning framework. We use momentum-based stochastic gradient descent (SGD) as the optimizer. For crack detection on a small dataset, 20 images, which are randomly selected from the training set, are used as the original samples. The models are fine-tuned for 5000 iterations, and we evaluate it every 200 iterations using the test set. To compare subsets generated by different data augmentation methods, we run the model training on each subset 3 times, respectively, and then average the maximum value of all runs to obtain the final average values for the augmentation methods.

### 4.4. Evaluation Method

#### 4.4.1. Evaluation Method of a Single Test Image

CovEval [16] is used to evaluate the performance of the crack detector. It is proposed to solve the problem of mean average precision (mAP), as the traditional evaluation [17] cannot fairly evaluate typical random fractal objects such as cracks. Specifically, CovEval uses cover area rate (CAr) instead of intersection over union (IoU) to calculate the box overlap. CAr (G,D) is formulated as:

(4) $$\mathrm{CAr}\left(G,D\right)=\frac{S_{G\cap D}}{\min\left(S_{G},\;S_{D}\right)}$$

where G and D represent a GT box and a detected box, respectively. $S_{G}$ and $S_{D}$ are areas of G and D, and $S_{G\cap D}$ is the intersection area of G and D.

Then extended recall (*XR*) and extended precision (*XP*) are used to represent the recall and precision; *XR* and *XP* are respectively defined as:

(5) $$XR=\frac{K_{r}}{n}$$

(6) $$XP=\frac{K_{p}}{m}$$

where *m* represents the number of detected boxes and *n* represents the noted GT boxes; $K_{r}$ and $K_{p}$ are the numbers of detected targets and valid detections, respectively.

The extended F-score ($F_{ext}$) is defined as:

(7) $$F_{ext}=\frac{2XP\times XR}{XP+XR}$$

Finally, the trade-off factor μ is defined and inserted into $F_{ext\;}$ to obtain the value of $F_{ext}^{\left(\mu \right)}$ as the key index of evaluation; $F_{ext}^{\left(\mu \right)}$ is computed as:

(8) $$F_{ext}^{\left(\mu \right)}=\frac{XP^{2\left(1-\mu \right)}\times XR^{2\mu }}{\left(1-\mu \right)XP+\mu XR}$$

It is suggested that the value of μ should be 0.8 in most defect detection tasks to make the recall (XR) dominant, because in practice, all defects must be detected to avoid catastrophic accidents. Further details regarding CovEval can be found in Li’s original paper. Following all the settings in their paper, in our study, we set μ as 0.8, the overlap threshold as 0.55, and the confidence threshold as 0.5.

#### 4.4.2. Evaluation Method for Inferencing Multiple Images

To evaluate the precision and recall of the model when stage two of our data augmentation is implemented, we treat identical cracks in multiple images as the same target. Therefore, we still have *n* noted GT boxes per image, and the number of detected boxes is expanded to the sum of $m_{1}$, $m_{2}$ … $m_{p}$ where the images to be detected are augmented for p−1 times.

Specifically, the modified extended recall (XR) and extended precision (XP) are defined. When there is at least one GT box corresponding to $D_{i}$, the detection is valid. Thus, the number of valid detection $K_{p}$ represents the sum of all valid detection boxes of one series of images, and XP can be defined as: XP=KpΣ1pmn.

Differently, if a GT box of the same crack in one series of images corresponds to any detected box, this means that this crack is successfully detected, and the data augmentation method is used to repeatedly detect the cracks and improve the XR. Therefore, the number of detected targets $K_{r}$ is counted in the series of images, and XR is defined as: $\mathrm{XR}=\frac{K_{r}}{n}$.

## 5. Results and Analysis

In this section, experiments are conducted to validate our proposed method. Firstly, the data augmentation methods in network training are tested to find the most effective ones, and, then, a crack detector is trained on an augmented small dataset. The second part discusses the greedy strategy for searching for the best data augmentation strategy in the inference process. Finally, the two-stage method is successfully applied to train good models with the small dataset (20 images).

### 5.1. Stage One: Data Augmentation in the Network Training

#### 5.1.1. Comparison of Data Augmentation Methods

First, we investigate different data augmentation methods of crack detection. Table 1 presents the results. It shows that nine data augmentation methods affect the performance of the model between −5.59% and 6.29%. Rotation is the most effective method to improve the performance of the model, and the value of $F_{ext}^{\left(0.8\right)}$ reaches 77.92%. However, even Gaussian blur results in an obvious degradation of the model by −5.59%.

Experiments of Gaussian blur and rotation show special results in crack detection. Studies show that the Gaussian blur can improve the generalization ability of CNN models in the recognition of natural object images [21,35], but it worsens the model for crack detection in the current experiments. According to the analysis in Section 3.1, the data augmentation method is dataset-specific. However, the crack is different from the general natural objects that the graphic feature of it is on a small scale. Obviously, the crack images processed by the Gaussian blur lose details, which will affect the perception of the model in regard to the features of the crack. Thus, this leads to a negative effect on the model. On the contrary, comparing with other data augmentation methods, the gain of rotation for the crack detection model is significant. This is because the directions of actual cracks are uncertain, and the samples in the small dataset cannot cover all the directions, while the rotation method generates crack images in various directions for CNN training without losing any graphic features. This is why the model can be significantly improved via rotation for augmentation. Although the other methods such as random crop and translation can generate crack images without losing any graphic features, the images generated via these methods also do not create valuable information for the model training, so they do not affect the performance of the model significantly. Because the rotation significantly improves the performance of the model, we delve into the method of rotation.

#### 5.1.2. The Rotation Method for Data Augmentation

In this subsection, we conduct four studies on the rotation method for crack detection, including four parts: a (rotation for augmenting datasets of different size); b (rotation with different augmentation factors); c (combining the rotation with other methods); d (rotation alleviates the over-fitting). The experiments follow the same setting as the previous mentioned, and the only difference is the maximum iterations of the CNN model. Note that all images are rotated by the angle of $\frac{\pi }{f}$, where f denotes the augmentation factor.

a. Rotation for Augmenting Datasets of Different Size.

**Figure 6:**
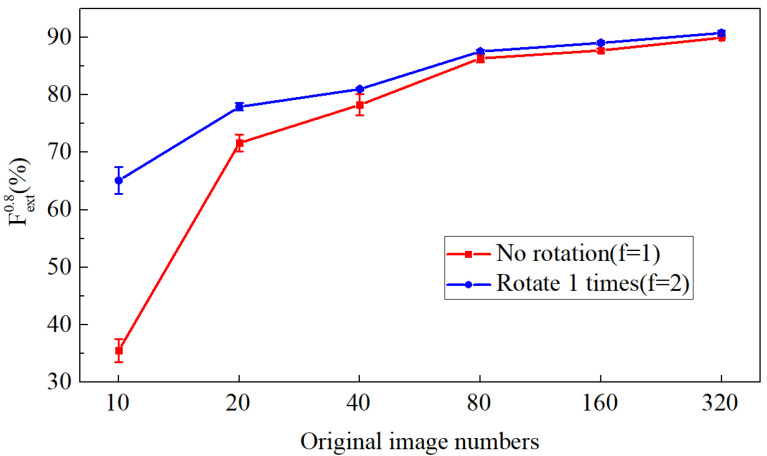
$F_{ext}^{\left(0.8\right)}$ of the models before and after rotation for different numbers of original images.

The rotation is applied to datasets with different numbers of samples for data augmentation. Figure 6 shows that the performance of the model is strongly related to the number of images used for network training in the case of crack detection. The more samples used in the training, the better the model. By data augmentation via rotation in experiments (take f=2, which means the augmentation doubles the samples), the models are all improved. Additionally, the higher the number of the original samples, the more limited the improvement of the model. Furthermore, the model trained with augmented images shows only slightly poor performance with the real sampled dataset, which includes the same number of images. This indicates that in some cases, the augmented images via rotation can even benefit the model for crack detection to the same extent as the real sampled images.

b. Rotation with Different Augmentation Factors.

**Figure 7:**
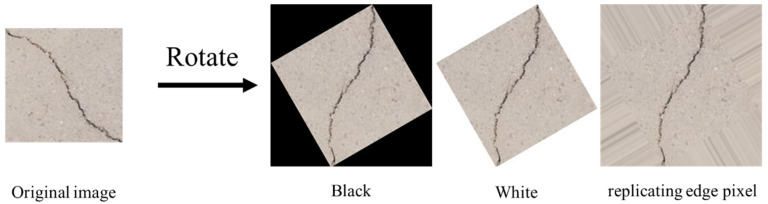
Three padding modes.

We discuss the effect of the data augmentation factor on the performance of the model trained with 20 original images for crack detection. Initially, it is necessary to identify the effect of different padding modes on the models during rotation when the rotated images are not straight (the rotation angle is not a multiple of π/4). We investigate three different image padding modes. Figure 7 shows the demos of these modes. The results presented in Table 2 indicate that replicating edge pixels can slightly improve the performance of model. In the following experiments, we adopt this image padding mode by default.

**Figure 8:**
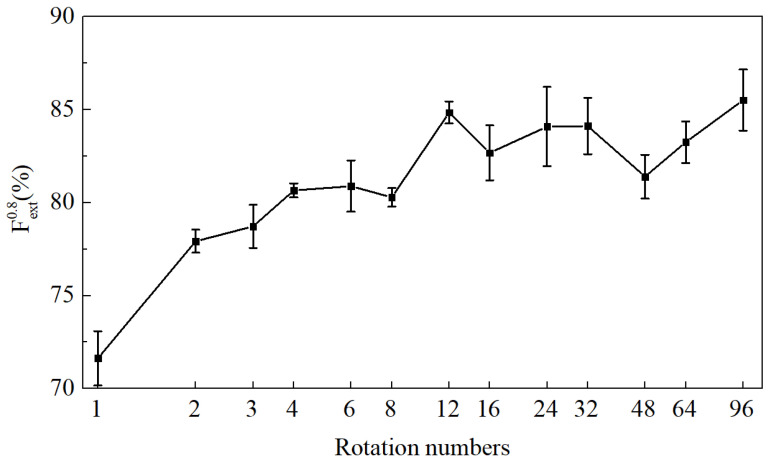
$F_{ext}^{\left(0.8\right)}$ of the model trained on datasets with different augmentation factors via rotation.

Figure 8 presents $F_{ext}^{\left(0.8\right)}$ of the trained models using different data augmentation factors for rotation. As we expected, with the increase in rotation times, the generalization performance improves significantly. Moreover, when the number of rotations is 96, $F_{ext}^{\left(0.8\right)}$ reaches saturation at 85.50%. Although the rotation is effective, the extra information provided by the augmentation is always limited. According to the experiments, the model trained on the augmented dataset from 20 original images performs just as well as when it is trained with 80 real sampled images.

c. Combining the Rotation with Other Methods.

For further improvement, we try to combine other three methods, which show good performance according to the results in Table 1, with the rotation method, respectively. Note that all the combined images are rotated before other transformations. As shown in Table 3, the results show that regardless of the combination of methods, the performance of the model is slightly decreased. This proves that rotation extracted the image information completely, and the added images augmented by other methods interfere with the network training, which results in performance degradation.

d. Rotation Alleviates the Over-Fitting.

**Figure 9:**
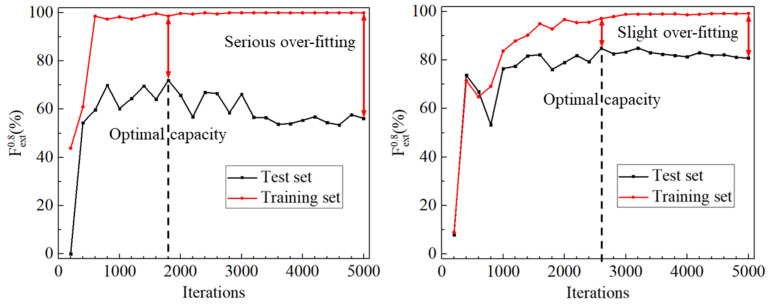
$F_{ext}^{\left(0.8\right)}$ of the model on the training and test set during the training iterations. The left figure is for training on the original dataset, and the right one is for training on the augmented dataset.

Over-fitting, which denotes the difference between the training error (on the training set) and the generalization error (on the test set), is a common phenomenon in training deep learning models. This issue is more serious for models trained on small datasets. We present the training error and the generalization error during the model training in Figure 9 to identify the influence of rotation on the over-fitting. According to the presented curves, the model appears to serious over-fitting when it is trained on only 20 original images. With the dataset augmented via rotation, this over-fitting is alleviated.

### 5.2. Stage Two: Data Augmentation in the Inference Process

For high-quality detection in practice, we propose stage two for the inference process. According to the results in Section 5.1, Table 1, the four best methods, namely, rotation, Gaussian noise, salt and pepper noise and vertical stretch, are selected as the candidates for augmentation in the inference. The constructed greedy strategy is shown in Table 4. Then, the greedy algorithm is used to search for the combination of data augmentation methods by rounds. Finally, the methods selected in every round comprise the most effective combination strategy. Note that the trained model used here is the best model trained on 20 original images after augmentation (rotate 96 times), and the maximum data augmentation in the inference process is set to 6.

**Figure 10:**
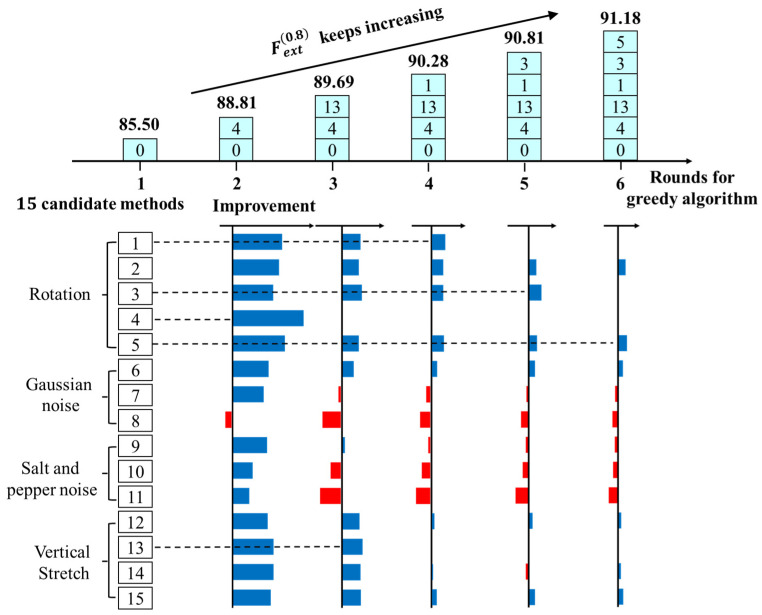
Search process and results of the greedy algorithm for the inference process. The rectangular box represents the candidate method, and the histogram indicates the improvement of the model when each method is added respectively. We select the method that obtains the maximum of the histogram in each round and add it to the final solutions being constructed. The sequence on the top shows the selected method added to the final solutions in different rounds by the greedy algorithm.

The results are shown in Figure 10. In every round, the remaining methods are applied to augment the images to be detected. According to the performance change, the method with the greatest improvement is selected and added into the combination. Among which, method 4 has the greatest performance improvement in the second round for the greedy search. Therefore, method 4 is added to the augmentation strategy in this round. When p is 6, we finally obtain the combination of augmentation methods after five rounds. Table 5 lists the details of all the selected methods. In addition, some conclusions can be drawn from the figure:

(1) Rotation is still the most effective data augmentation method in the inference process, and rotations with different angles impact the model in slightly different manners.
(2) Gaussian noise and salt and pepper noise are not good for improving the model in the inference process, and they even cause a negative influence in some cases.
(3) When there is a stretch method in the constructed solutions, the model can gain little improvement by adding other stretch methods.

### 5.3. Two-Stage Method for Network Training within 20 Images

**Figure 11:**
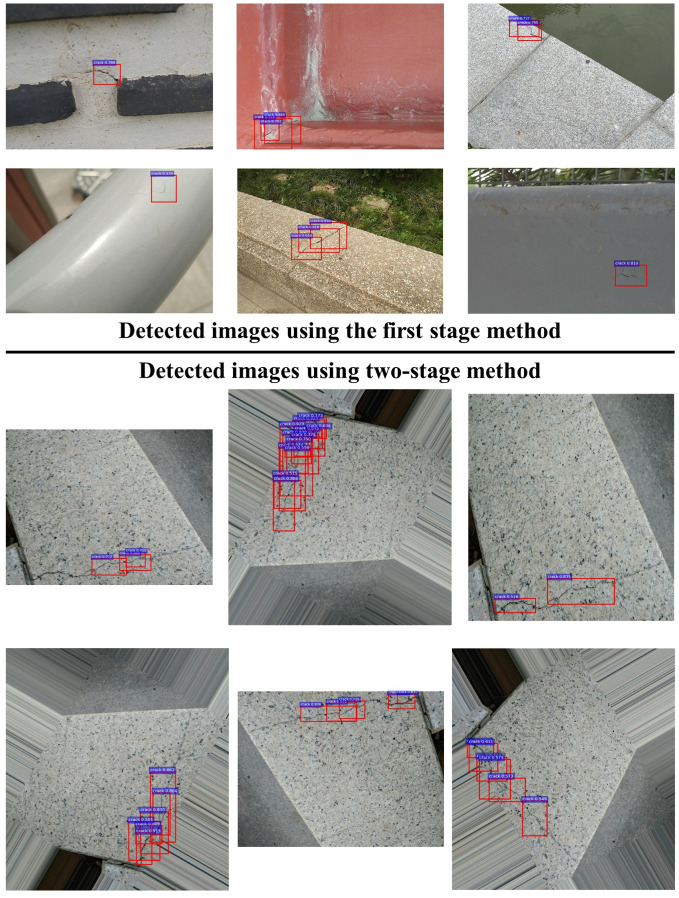
Detected crack images with red boxes. The images above the horizontal line are the examples of crack detection only using the first-stage method. The images below the horizontal line are the examples of crack detection using the two-stage method. By augmenting the image to be detected into a series of augmented images, the trained model has more chances to detect and identify any missing targets when processing an image.

Integrating the proposed two-stage method, 20 images are rotated 96 times for data augmentation in network training. Then, in the inference process, six images generated by the searched combination of the augmentation methods are used for detecting the cracks. The trained model performs better via a repetitive detection on these augmented images. As seen from Table 6, the $F_{ext}^{\left(0.8\right)}$ is as high as 91.18%, and the extended recall (XR) reaches 96%, meaning that it can meet the requirements of some industrial applications. Compared with the model trained with 20 original images, the $F_{ext}^{\left(0.8\right)}$ and XR of the model increase by 27.29% and 38.81%, respectively. Compared with Li’s work, with 320 real sampled images for training, its $F_{ext}^{\left(0.8\right)}$ and XR obtain 91.64% and 93.6%, respectively. When using the two-stage method for data augmentation, our model achieves a competitive performance with only 20 original images for training. Thus, this implies that we achieve a great improvement in crack detection with small datasets. Figure 11 shows the demo-detected images from our best model.

## 6. Conclusions

In this paper, a two-stage data augmentation method for crack detection is proposed. Several methods for data augmentation are studied in the model training and inference process for better performance with the limited dataset. In stage one, the greatest improvement is obtained by the image rotation among nine methods for data augmentation. With the best setting, the model for crack detection is improved significantly when adopting the rotation for data augmentation. Moreover, the data augmentation also alleviates the over-fitting in network training. In stage two, we also adopt the data augmentation method in the inference process of the trained models. The greedy algorithm is used to search for the most effective combination in this performance–source trade-off. With the two-stage method, the model based on deep learning for crack detection is effectively trained and applied based on a small dataset of 20 samples. The trained model shows an applicable performance whose $F_{ext}^{\left(0.8\right)}$ reaches 91.18%, and the recall of crack (XR) reaches 96%.

## Figures and Tables

**Table 1:** $\;F_{ext}^{\left(0.8\right)}$ of different data augmentation methods of crack detection. The number of original images is 20, and the data augmentation factor f is 2.

	Method	Case	$F_{ext}^{\left(0.8\right)}$ (%)	Relative Change (%)
	Baseline		71.63 ± 1.46	
Geometric transformation	Stretch	Horizontal	71.52 ± 1.00	−0.11
		Vertical	74.66 ± 1.73	+3.03
	Random crop		72.24 ± 1.87	+0.61
	Translation		69.94 ± 1.80	−1.69
	Rotation		77.92 ± 0.63	+6.29
Photometric transformation	Gamma transformation	Slight	71.91 ± 2.81	+0.28
		Strong	73.34 ± 0.98	+1.71
	Gaussian blur	Slight	66.84 ± 1.97	−4.79
		Strong	66.04 ± 3.29	−5.59
	Gaussian noise	Slight	72.06 ± 1.49	+0.43
		Strong	75.42 ± 1.06	+3.79
	Salt and pepper noise	Slight	75.34 ± 1.56	+3.71
		Strong	71.82 ± 3.18	+0.19
	Histogram equalization		72.25 ± 1.92	+0.62

**Table 2:** $F_{ext}^{\left(0.8\right)}$ of the models trained with rotation for data augmentation adopting different padding mode. Twenty original images are used, and the data augmentation factor f is 3.

Method	$F_{ext}^{\left(0.8\right)}$ (%)	Relative Changes (%)
Black	78.40 ± 1.68	0
White	77.96 ± 2.36	−0.44
Replicating edge pixel	78.72 ± 1.16	+0.32

**Table 3:** $F_{ext}^{\left(0.8\right)}$ of the models trained on datasets augmented by different combined methods. Twenty original images are used, and the data augmentation factor f is 96.

Augmentation Method	$F_{ext}^{\left(0.8\right)}$ (%)	Relative Changes (%)
Rotation	85.50 ± 1.64	0
Rotation + salt and pepper noise	83.96 ± 0.27	−1.54
Rotation + Gaussian noise	83.36 ± 1.07	−2.14
Rotation + stretch	84.54 ± 0.35	−0.96
Rotation + salt and pepper noise + stretch	83.79 ± 1.17	−1.71
Rotation + Gaussian noise + stretch	82.94 ± 1.37	−2.56

**Table 4:** List of the candidate augmentation methods and the implementation details.

Method	Case	Number
Rotation	Rotate 60°	1
	Rotate 120°	2
	Rotate 180°	3
	Rotate 240°	4
	Rotate 300°	5
Gaussian noise (m is the mean, σ is the standard deviation)	m = 0 σ = 10	6
	m = 0 σ = 20	7
	m = 0 σ = 30	8
Salt and pepper noise (*p* is the probability of random salt and pepper noise)	*p* = 0.025	9
	*p* = 0.0375	10
	*p* = 0.05	11
Vertical Stretch	Stretch 1.25 times	12
	Stretch 1.5 times	13
	Stretch 1.75 times	14
	Stretch 2 times	15

**Table 5:** Search results of the greedy algorithm in the inference process.

Rounds of the Greedy Algorithm	Specific Strategies	$F_{ext}^{\left(0.8\right)}$ (%)
1	No augmentation	85.50
2	Rotate 240°	88.81
3	Rotate 240°, stretch 1.5 times	89.69
4	Rotate 240°, 60°, respectively, stretch 1.5 times	90.28
5	Rotate 240°, 60°, 180°, respectively, stretch 1.5 times	90.81
6	Rotate 240°, 60°, 180°, 300°, respectively, stretch 1.5 times	91.18

**Table 6:** $F_{ext}^{\left(0.8\right)}$ and extended recall (XR) of different methods of crack detection.

Method	Number of Training Set	$F_{ext}^{\left(0.8\right)}$ (%)	XR(%)
Li’s paper	320	91.64	93.6
No augmentation (ours)	20	71.63	69.16
First-stage method (ours)	20	85.50	88.91
Two-stage method (ours)	20	91.18	96
